# First record of *Ibidoecus plataleae* (Denny, 1842) (Ischnocera: Philopteridae) from the Black-faced Spoonbill, *Platalea minor* Temminck and Schlegel, 1849 (Pelecaniformes: Threskiornithidae) in the Republic of Korea

**DOI:** 10.1016/j.ijppaw.2026.101269

**Published:** 2026-07-31

**Authors:** Jeong Hun Shim, Heon Woo Lee, Min-Seung Yang, In-Ki Kwon, Yeong-Deok Han, Seongjun Choe

**Affiliations:** a Department of Parasitology, School of Medicine, Chungbuk National University, Cheongju, 28644, Republic of Korea; b Research Center for Endangered Species, National Institute of Ecology, Yeongyang, 36531, Republic of Korea; c Waterbird Network Korea, Seoul, 01795, Republic of Korea; d Bird Research SaeZiP, Yangpyeong, 12504, Republic of Korea

**Keywords:** Bird lice, Chewing louse, Ectoparasites, Endangered bird species, Phthiraptera

## Abstract

Chewing lice are highly host-specific, permanent ectoparasites of birds. This feature potentially places lice infesting endangered hosts at an even greater risk of extinction than the hosts themselves. Despite this ecological significance, research on chewing lice parasitizing endangered birds in the Republic of Korea remains limited. This study aims to document and provide taxonomic information on the chewing lice species infesting the wild population of the endangered Black-faced Spoonbill (*Platalea minor*) in Korea. In July 2025, five spoonbills were examined for ectoparasites. Collected specimens were morphologically identified using light microscopy and scanning electron microscopy (SEM), focusing on key diagnostic characters. Additionally, we performed a phylogenetic analysis and compared genetic distances. Based on the morphological and molecular analyses, our specimens were identified as *Ibidoecus plataleae* (Denny, 1842). This study represents the first report of *I*. *plataleae* from the Black-faced Spoonbill in Korea.

## Highlights

• New record of *Ibidoecus plataleae* from the Black-faced Spoonbill (*Platalea minor*) in Republic of Korea.
• Precise identification of diagnostic morphological features using light microscopy analysis.
• Provides baseline morphometric data for *I. plataleae.*
• Scanning electron microscopy provides a detailed habitus of *I. plataleae.*
• Phylogenetic analysis and genetic distance assessment of *I. plataleae.*

## Graphical abstract

**Figure:**
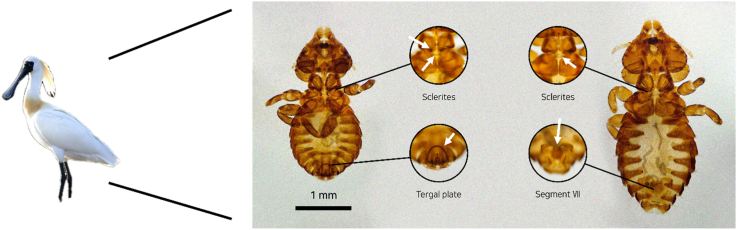

## 1 Introduction

Chewing lice (Phthiraptera: Ischnocera and Amblycera) are permanent ectoparasites of birds and mammals that feed on dead skin, feathers, and secretions (Price et al., 2003; Clayton et al., 2007). These ectoparasites spend their entire life cycle on the host's body, resulting in high host specificity which potentially drives lice on endangered hosts to face an extinction risk even greater than that of their hosts (Price et al., 2003; Clayton et al., 2007; Pizzi, 2009; Shim et al., 2026). Indeed, *Colpocephalum californici* Price & Beer, 1963 went extinct during antiparasitic treatments implemented as part of the breeding program for the California Condor, *Gymnogyps californianus* (Shaw, 1797) (Pizzi, 2009). Thus, to prevent unintended extinctions, it is necessary to pay close attention not only to endangered hosts but also to their associated species, including ectoparasites. Nevertheless, with the exception of the Oriental Stork, *Ciconia boyciana* Swinhoe, 1873 and the Tundra Swan, *Cygnus columbianus* (Ord, 1815), systematic investigations into chewing lice on avian species designated as endangered by the Ministry of Climate, Energy and Environment of Korea have been limited in the Republic of Korea (Noh, 1984; Shim et al., 2026).

The Black-faced Spoonbill, *Platalea minor* Temminck and Schlegel, 1850 (Pelecaniformes: Threskiornithidae), is designated as an ‘Endangered wildlife Class Ⅰ' by the Ministry of Climate, Energy and Environment of Korea and is an endemic species of East Asia (Kwon et al., 2015; Shimada and Yoshizawa, 2019; Yoo et al., 2024). Approximately 93% of the global population of this species is known to breed along the western coast of the Korean Peninsula. To protect these birds, various studies have been conducted in Korea, including research on nest site characteristics and captive rearing (Kwon et al., 2015; Yoo et al., 2024). However, no studies have yet been conducted in Korea on the ectoparasites that interact with and affect this endangered bird species.

Investigations into chewing lice parasitizing the Black-faced Spoonbill have been conducted in Japan, where three louse species (*Ardeicola plataleae* (Linnaeus, 1758), *Eucolpocephalum femorale* (Piaget, 1880), and *Ibidoecus plataleae* (Denny, 1842)) were reported (Shimada and Yoshizawa, 2019). Considering the migratory habits of the spoonbill (Kwon et al., 2015; Chen et al., 2021), it is expected that the chewing lice community in the Korean population will be identical to that found in Japan. Therefore, this study aimed to investigate and identify the chewing lice communities of the Black-faced Spoonbill in Korea for the first time.

This is the first study of a chewing louse species found in the wild population of the Black-faced Spoonbill in Korea, a region that has remained unstudied despite being a core breeding site. Specifically, this study provides comprehensive morphometric data, scanning electron microscope (SEM) images, and genetic information for this single species.

## 2 Materials & methods

### 2.1 Sampling and morphological analysis

Field sampling was conducted at the Namdong Reservoir in the Republic of Korea, a breeding site for the Black-faced Spoonbill. This study was performed under the approval of the National Institute of Ecology (Approval No. NIE-B-2025-46). On July 5, 2025, five individuals of the Black-faced Spoonbill were examined for chewing lice. Lice were collected via visual examination and manual picking using fine forceps. The collected lice were immediately preserved in 70% ethanol. To observe detailed morphological characteristics, eight specimens were cleared in a 10% KOH solution and mounted on glass slides using Hoyer's medium. The mounted specimens were observed under an Olympus CX33 light microscope (Olympus, Tokyo, Japan). The remaining specimens were photographed using a JSM-6390LV SEM(JEOL, Japan). Morphological identification and taxonomic diagnosis were performed based on the diagnostic keys and descriptions provided in previous studies (Denny, 1842; Tandan, 1958; Shimada and Yoshizawa, 2019).

### 2.2 DNA sequencing and molecular analysis

Genomic DNA (gDNA) was extracted from two specimens of chewing lice using the QIAamp DNA Micro Kit (QIAGEN, Hilden, Germany). To amplify the mitochondrial cytochrome *c* oxidase subunit Ⅰ (COⅠ) fragment, the primer set L6625 (5′-CCGGATCCTTYTGRTTYTTYGGNCAYCC-3′) and H7005 (5′-CCGGATCCACNACRTARTANGTRTCRTG-3′) was employed (Hafner et al., 1994). Polymerase chain reaction (PCR) was performed using EzPCR™ XO 5X Master Mix (ELPIS-Biotech, Daejeon, Korea) under the following cycling conditions: an initial denaturation at 95 °C for 3 min; 40 cycles of 95 °C for 30 s, 56 °C for 20 s, and 72 °C for 30 s; and a final extension at 72 °C for 5 min. PCR amplicons were sequenced via Sanger sequencing at Cosmogenetech (Daejeon Branch, Republic of Korea). Sequence editing was conducted using GENEIOUS v.2025.2.2 software, resulting in COⅠ gene fragments ranging from 413 to 436 bp. For the phylogenetic analysis, a total of 40 COⅠ sequences were utilized, including two newly generated sequences from this study and 38 sequences retrieved from the National Center for Biotechnology Information (NCBI) database (35 from the family Philopteridae and three mammalian lice species as an outgroup). Except for the genus *Ibidoecus*, the remaining philopterid sequences were obtained by selecting one random sequence per species from the closely related genera identified in Johnson et al. (2012).

Sequence editing for phylogenetic analysis was performed using GENEIOUS, and the COⅠ fragments were subsequently trimmed to a uniform length of 295 bp. A phylogenetic tree for the 40 lice species (Table 1) was constructed using the maximum likelihood (ML) algorithm in PhyML v.3.0 (Guindon et al., 2010). The general time reversible (GTR) + gamma distribution and invariant sites (G + I) model was selected as the optimal nucleotide substitution model based on Smart Model Selection (SMS) (Lefort et al., 2017). The robustness of the phylogenetic nodes was evaluated using 1000 bootstrap replicates (Felsenstein, 1985). Additionally, genetic similarity and pairwise *p*-distances for species of the genus *Ibidoecus* were calculated using GENEIOUS and Mega X v. 12.0.15 software, respectively, to assess their genetic relationships (Table 1).

**Table 1:** Chewing lice species used for molecular analysis in this study (COⅠ fragment).

Lice species	Avian host species	Sample source	GenBank accession No.	Reference
*Anaticola anseris*	*Branta canadensis*	-	DQ314501	Johnson et al. (2006)
*Anaticola asymmetricus*	*Alopochen aegyptiaca*	Republic of South Africa	KT587824	Escalante et al. (2016)
*Anaticola australis*	*Cereopsis novaehollandiae*	Australia	KT587854	Escalante et al. (2016)
*Anaticola branderi*	*Clangula hyemalis*	United States of America	KT587859	Escalante et al. (2016)
*Anaticola constrictus*	*Melanitta perspicillata*	United States of America	KT587803	Escalante et al. (2016)
*Anaticola crassicornis*	*Mareca sibilatrix*	Argentina	DQ007339	Covacin et al. (2006)
*Anaticola magnificus*	*Tadorna tadornoides*	-	DQ314500	Johnson et al. (2006)
*Anaticola marginella*	*Chloephaga picta*	Falkland Islands (Islas Malvinas)	KT587858	Escalante et al. (2016)
*Anaticola mergiserrati*	*Malacorhynchus membranaceus*	Australia	KT587860	Escalante et al. (2016)
*Anaticola rheinwaldi*	*Branta bernicla*	Sweden	KT587850	Escalante et al. (2016)
*Anaticola tamarae*	*Histrionicus histrionicus*	United States of America	KT587823	Escalante et al. (2016)
*Anatoecus dentatus*	*Cygnus atratus*	China	ON585119	Unpublishied
*Anatoecus icterodes*^a^	-	-	ON643885	Sweet et al. (2022)
*Docophoroides brevis*	-	-	ON643912	Sweet et al. (2022)
*Forficuloecus banksi*	*Psephotus varius*	Australia	EU669827	Price et al. (2008)
*Forficuloecus cameroni*	*Aprosmictus erythropterus*	Australia	EU669830	Price et al. (2008)
*Forficuloecus forficula*	*Platycercus elegans*	Australia	EU669825	Price et al. (2008)
*Forficuloecus josephi*	*Neopsephotus bourkii*	Australia	EU669829	Price et al. (2008)
*Forficuloecus palmai*	*Barnardius zonarius*	Australia	EU669828	Price et al. (2008)
*Forficuloecus pezopori*	*Pezoporus flaviventris*	Australia	PP524917	Martin et al. (2024)
*Forficuloecus wilsoni*	*Platycercus venustus*	Australia	EU669826	Price et al. (2008)
*Harrisoniella densa*	*Phoebastria immutabilis*	-	AF545712	Johnson et al. (2003)
*Harrisoniella ferox*	*Thalassarche melanophris*	-	AF396568	Johnson et al. (2003)
*Harrisoniella hopkinsi*	*Diomedea epomophora*	-	AF396569	Johnson et al. (2003)
*Ibidoecus bisignatus*	*Plegadis chihi*	-	AY314817	Smith et al. (2004)
*Ibidoecus bisignatus*	-	-	JN122005	Cameron et al. (2011)
*Ibidoecus bisignatus*	-	-	ON643931	Sweet et al. (2022)
*Ibidoecus meinertzhageni*	*Nipponia nippon*	China	OK032412	Unpublishied
*Ibidoecus meinertzhageni*	*Nipponia nippon*	China	OK032413	Unpublishied
*Ibidoecus meinertzhageni*	*Nipponia nippon*	China	OK032414	Unpublishied
*Ibidoecus meinertzhageni*	*Nipponia nippon*	China	OK032415	Unpublishied
*Ibidoecus meinertzhageni*	*Nipponia nippon*	China	OK032416	Unpublishied
*Ibidoecus meinertzhageni*	*Nipponia nippon*	China	OK032417	Unpublishied
*Ibidoecus plataleae*	*Platalea leucorodia*	China	ON585570	Cao et al. (2024)
*Ibidoecus plataleae*	*Platalea minor*	Republic of Korea	**PZ676668**	**This study**
*Ibidoecus plataleae*	*Platalea minor*	Republic of Korea	**PZ676669**	**This study**
*Psittoecus eos*	*Cacatua sanguinea*	-	JX121677	Johnson et al. (2012)
*Bovicola bovis*	Outgroup (Mammals)	-	AF545680	Johnson et al. (2003)
*Stachiella larseni*	Outgroup (Mammals)	-	JX121679	Johnson et al. (2012)
*Trichodectes octomaculatus*	Outgroup (Mammals)	-	AY314823	Smith et al. (2004)

a Currently synonymized with *Anatoecus dentatus* (Grossi et al., 2014).

## 3 Results

### 3.1 Morphological analysis

Parvorder Ischnocera Kellogg, 1896.

Family Philopteridae Burmeister, 1838

**Fig. 1:**
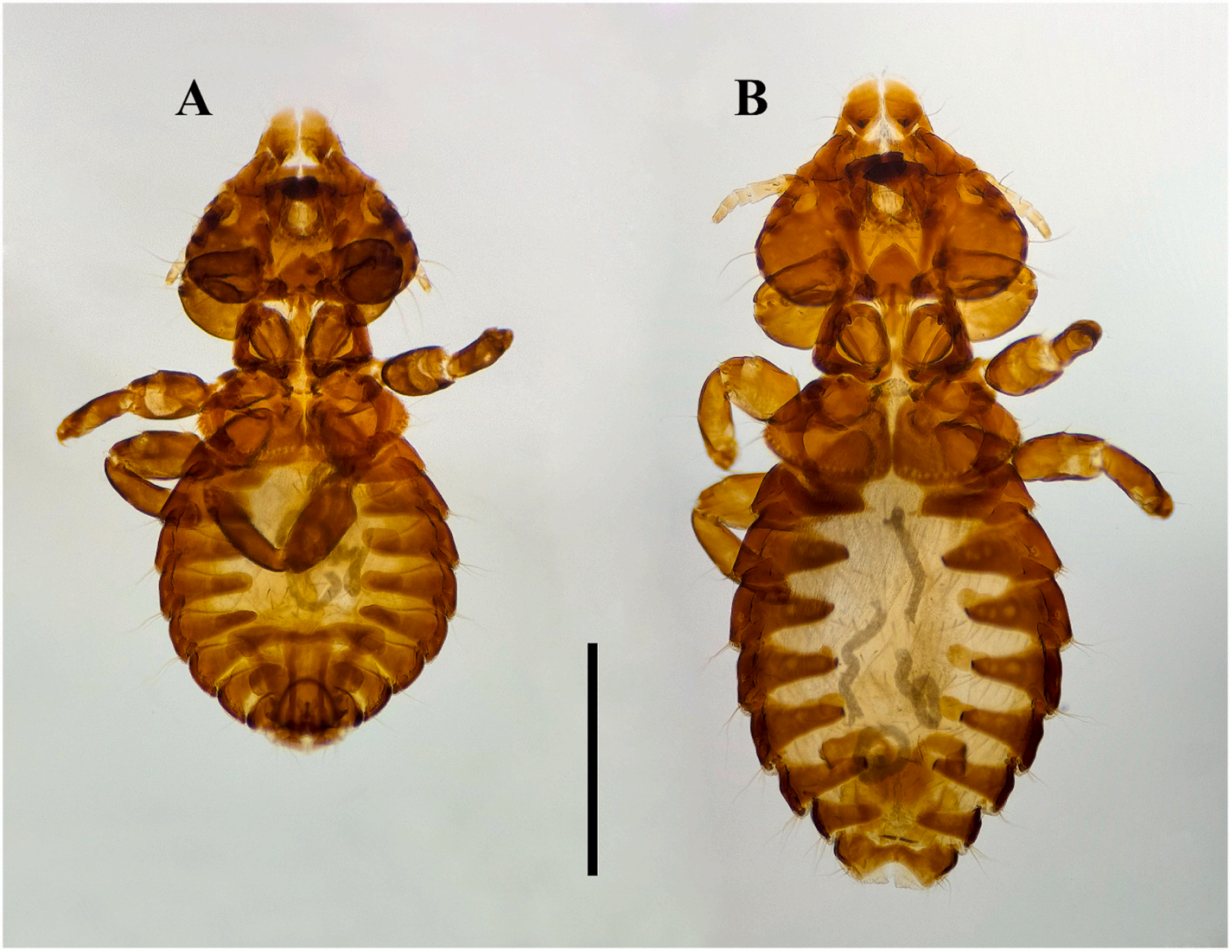
Dorsal view of *Ibidoecus plataleae* with a 1 mm scale bar. (A) Male. (B) Female.

**Fig. 2:**
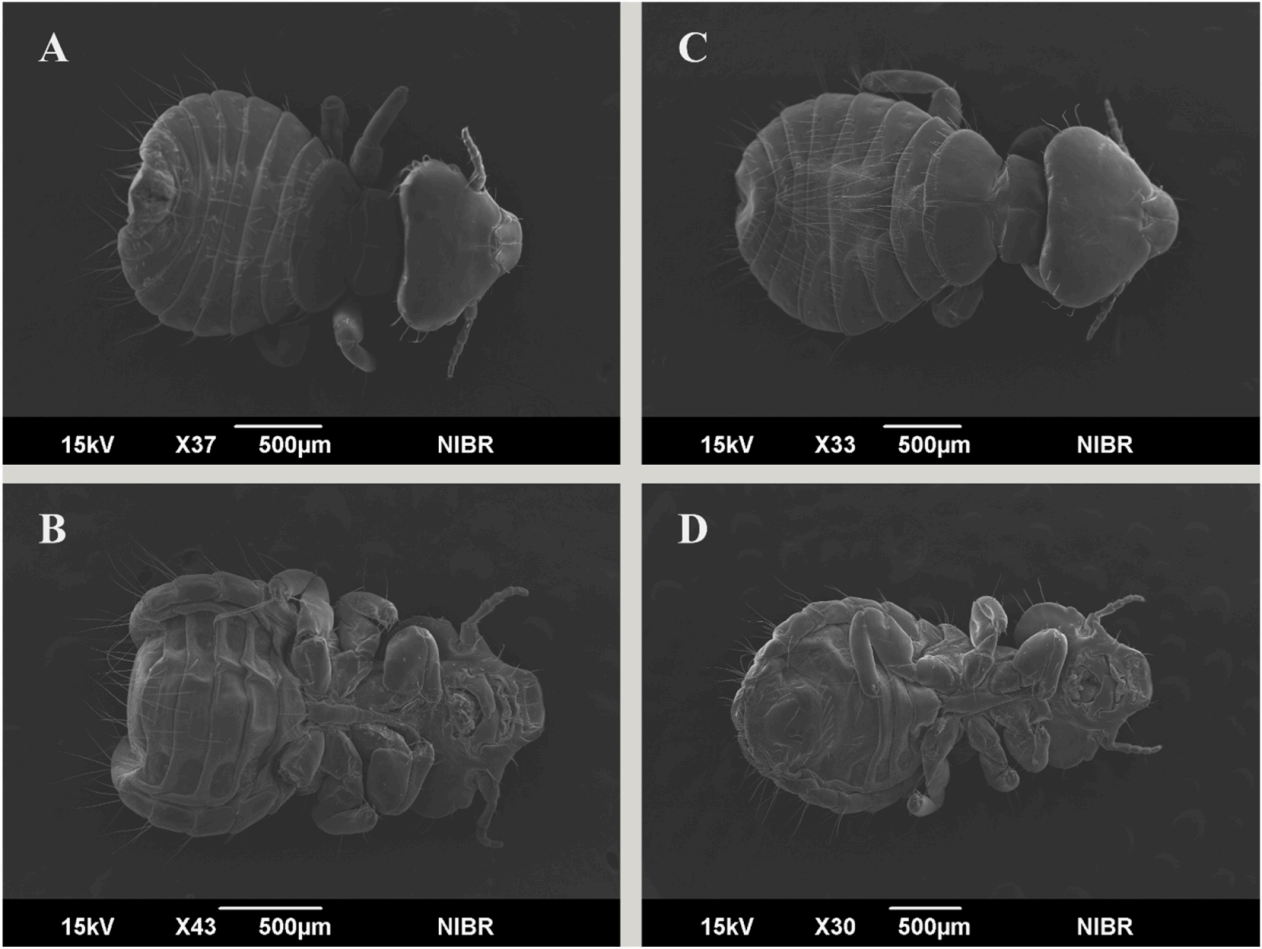
Scanning electron micrographs of *Ibidoecus plataleae*. (A–B) Male: (A) dorsal view; (B) ventral view. (C–D) Female: (C) dorsal view; (D) ventral view.

**Fig. 3:**
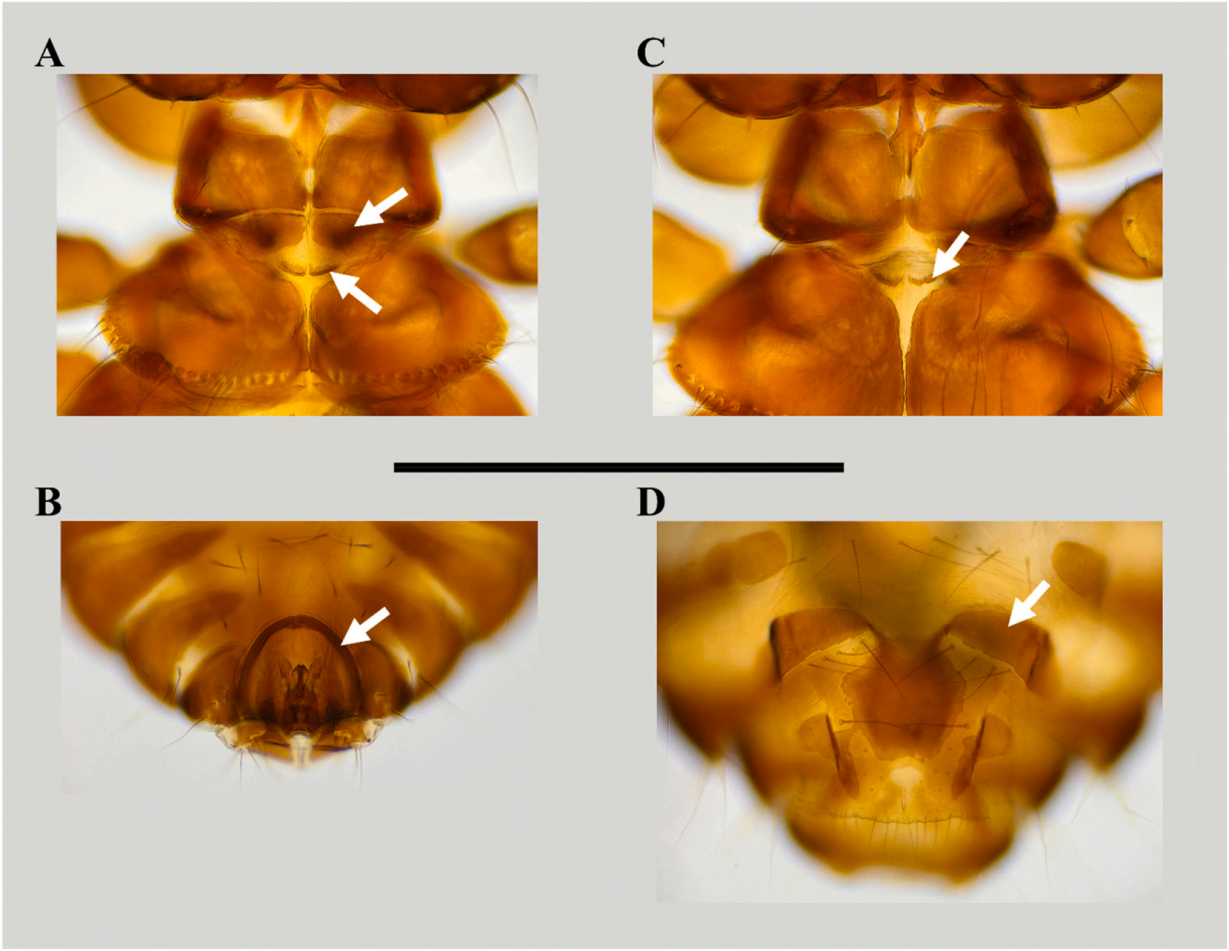
Details of *Ibidoecus plataleae* with a 1 mm scale bar. (A–B) Male: (A) sclerites between pronotum and pteronotum; (B) anterior tergal plate. (C–D) Female: (C) sclerites between pronotum and pteronotum; (D) lateral sternal plate on segment Ⅶ.

**Fig. 4:**
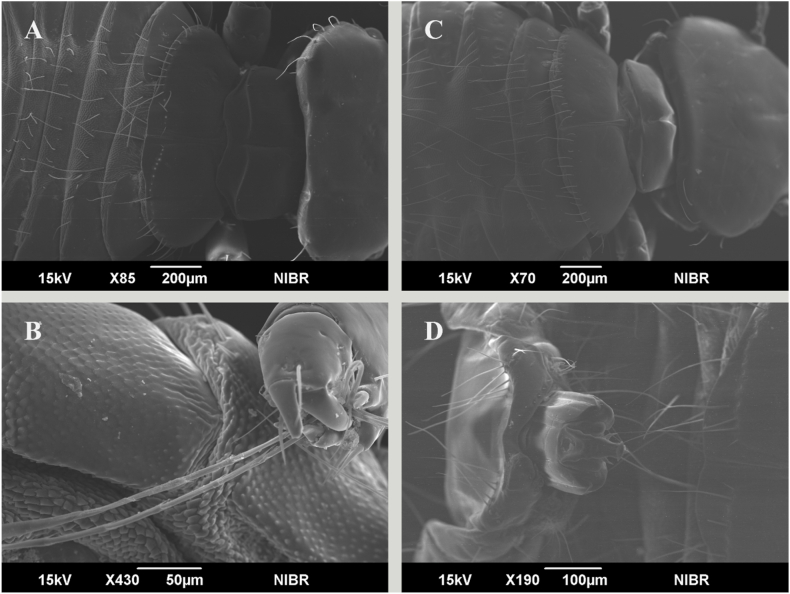
Scanning electron micrographs showing morphological details of *Ibidoecus plataleae*. (A) Male pronotum and pteronotum. (B) Male leg Ⅲ claw grasping a feather barb. (C) Female pronotum and pteronotum. (D) Male genitalia.

***Ibidoecus plataleae*** (Denny, 1842) **(**Fig. 1, Fig. 2, Fig. 3, Fig. 4).

저어새털이(신칭).

*Docophorus plataleae* Denny (1842): 100-101, plate Ⅳ, Fig. 9.

**Fig. 5:**
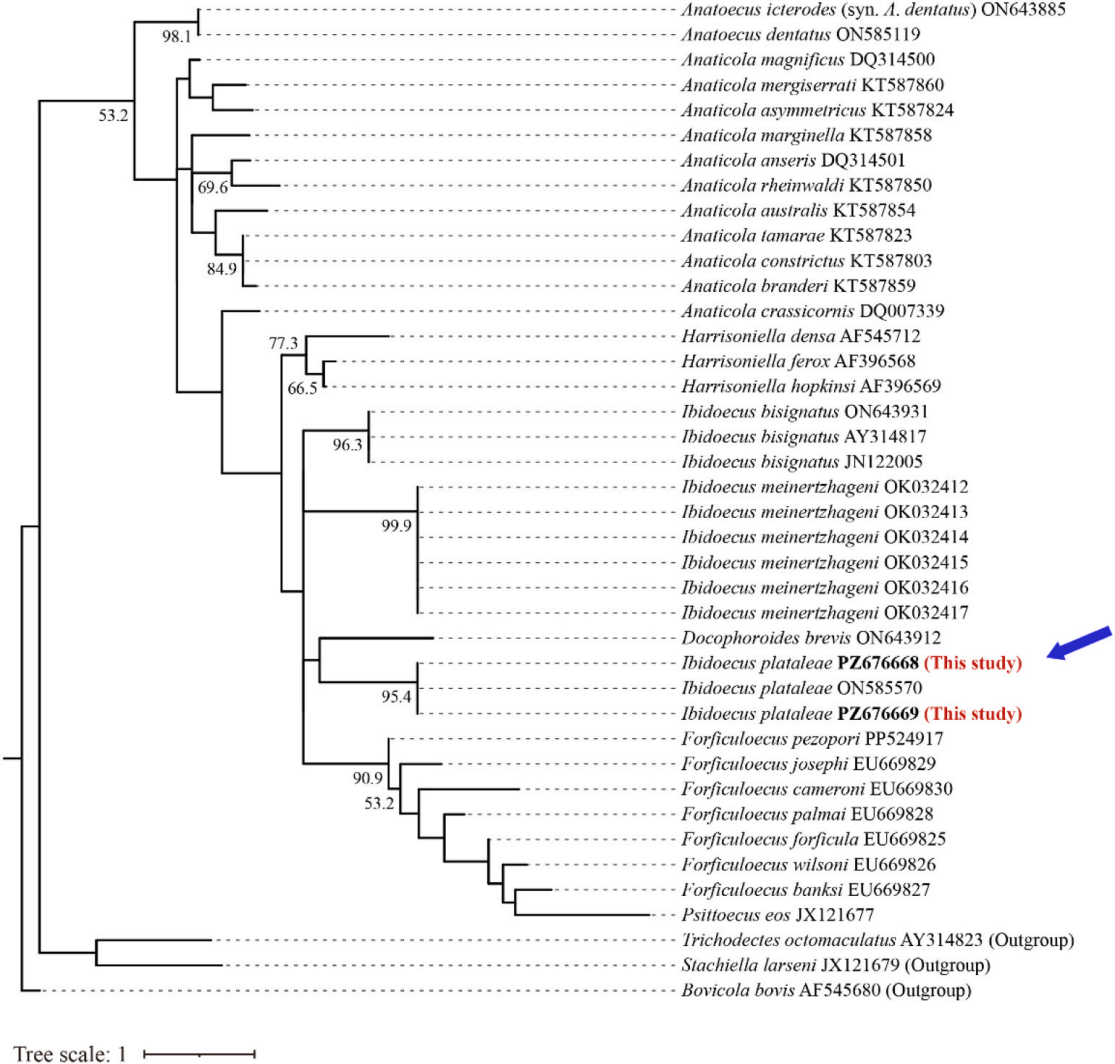
ML phylogenetic tree of the genus *Ibidoecus* within Philopteridae based on COⅠ partial sequences (295 bp). Numbers at nodes indicate bootstrap values (>50%).

*Docophorus sphenophorus* Nitzsch, in Giebel (1874): 99, tabula Ⅻ, Fig. 4; Piaget, 1880: 89, plate Ⅶ, Fig. 5.

*Ibidoecus plataleae*: Uchida, 1948: 311; Tandan (1958): 406-407, Fig. 5, 9, 22-26; Shimada and Yoshizawa (2019): 134, Fig. 1a, b, 2a.

**Material examined.** 4 males and 4 females (NIBRIN0001101590-NIBRIN00011015907) from the Black-faced Spoonbill, *Platalea minor* Temminck and Schlegel, 1849 (Pelecaniformes: Threskiornithidae), the Republic of Korea, Namdong Reservoir (37°23′27″N, 126°40′38″E), July 5, 2025, collected by Han Y.-D.

**Diagnosis.** In both sexes: Head and thorax deep chestnut color; abdomen nearly orbicular, with elongate liver-coloured fasciae; head obtusely triangular, with two liver-coloured bands; anterior dorsal plates and preantennal region short; posterodorsal marginal setae, extended to midline of divided pteronotum (Fig. 1, Fig. 2). In males: total length 2.56-2.83 mm, greatest width 1.26-1.52 mm, cephalic index 1.28-1.32 (Table 2); two sclerites, one large and one small, present between pronotum and pteronotum each side (Fig. 3A); 17-21 posterodorsal marginal setae on each side (Fig. 4A); heavily sclerotized and semicircular anterior tergal plate on terminal segment of abdomen (Fig. 3B). In females: total length 3.41-3.59 mm, greatest width 1.58-1.67, cephalic index 1.23-1.29 (Table 2); one small sclerites, present between pronotum and pteronotum each side (Fig. 3C); 16-21 posterodorsal marginal setae on each (Fig. 4C); inner margin of each lateral sternal plate on segment Ⅶ joined to the central portion of genital plate (Fig. 3D).

Remarks. I. plataleae was originally described from its type host, the Eurasian Spoonbill (Platalea leucorodia). According to the original description, its abdominal fasciae vary considerably in both color and extent (Denny, 1842).

**Table 2:** Morphometric comparison of *Ibidoecus plataleae* including minimum and maximum values by host species.

Measurements	Eurasian spoonbill (Tandan, 1958)		Black-faced spoonbill (This study)	
	Male (9)	Female (13)	Male (4)	Female (4)
Total length (mm)	-	-	2.56-2.83	3.41-3.59
Greatest width (mm)	-	-	1.26-1.52	1.58-1.67
Cephalic index (%)	1.21-1.31	1.20-1.35	1.28-1.32	1.23-1.29

In this study, we provide an improved diagnosis of *I*. *plataleae* to prevent potential confusion for future researchers. For example, referencing only the most recent study by Shimada and Yoshizawa (2019) might result in misidentifying the species because of the variation in the number of posterodorsal marginal setae. Shimada and Yoshizawa (2019) reported 17 and 19 setae on each side in males and females, respectively. However, we observed clear intra-individual variation in this study: the male had 19 left and 21 right setae (Fig. 4A), and the female had 19 left and 17 right setae (Fig. 4C). Furthermore, this study offers baseline morphometric data for *I. plataleae* (Table 2), providing necessary data for the future population conservation of this species.

### 3.2 Molecular analysis

In the ML phylogenetic tree constructed using a 295 bp COⅠ fragment, our two *I*. *plataleae* sequences were grouped with the previously registered *I*. *plataleae* sequence, exhibiting a 95.4% bootstrap support for this grouping (Fig. 5). Based on a 378 bp COⅠ fragment, the genetic distance among the three *I*. *plataleae* sequences showed 99.74-100% genetic similarity and a *p*-distance of 0% (Table 3). The genetic distance between *I*. *plataleae* and other *Ibidoecus* spp. ranged from 72.75 to 75.93% in genetic similarity and 28.48-33.47% in *p*-distance (Table 3).

**Table 3:** Genetic distances among 12 *Ibidoecus* species based on COⅠ partial sequences (378 bp). Values (%) below the diagonal represent genetic similarity, and those above the diagonal indicate pairwise p-distances.

	AY314817	JN122005	ON643931	OK032412	OK032413	OK032414	OK032415	OK032416	OK032417	ON585570	BS1F	BS2M
*I*. *bisignatus* AY314817	-	0.00	0.00	24.51	24.51	24.51	24.51	24.51	24.51	28.51	28.48	28.48
*I*. *bisignatus* JN122005	100.00	-	0.00	24.51	24.51	24.51	24.51	24.51	24.51	28.51	28.48	28.48
*I*. *bisignatus* ON643931	100.00	100.00	-	24.51	24.51	24.51	24.51	24.51	24.51	28.51	28.48	28.48
*I*. *meinertzhageni* OK032412	78.31	78.31	78.31	-	0.00	0.00	0.00	0.00	0.00	33.47	33.42	33.42
*I*. *meinertzhageni* OK032413	78.31	78.31	78.31	100.00	-	0.00	0.00	0.00	0.00	33.47	33.42	33.42
*I*. *meinertzhageni* OK032414	78.31	78.31	78.31	100.00	100.00	-	0.00	0.00	0.00	33.47	33.42	33.42
*I*. *meinertzhageni* OK032415	78.31	78.31	78.31	100.00	100.00	100.00	-	0.00	0.00	33.47	33.42	33.42
*I*. *meinertzhageni* OK032416	78.31	78.31	78.31	100.00	100.00	100.00	100.00	-	0.00	33.47	33.42	33.42
*I*. *meinertzhageni* OK032417	78.31	78.31	78.31	100.00	100.00	100.00	100.00	100.00	-	33.47	33.42	33.42
*I*. *plataleae* ON585570	75.66	75.66	75.66	72.75	72.75	72.75	72.75	72.75	72.75	-	0.00	0.00
*I*. *plataleae* **BS1F**	75.93	75.93	75.93	73.02	73.02	73.02	73.02	73.02	73.02	99.74	-	0.00
*I*. *plataleae* **BS2M**	75.93	75.93	75.93	73.02	73.02	73.02	73.02	73.02	73.02	99.74	100.00	-

## 4 Discussion

This study conducted a morphological and molecular analysis of *Ibidoecus plataleae*, providing an enhanced diagnosis by integrating data from earlier literature (Denny, 1842; Tandan, 1958; Shimada and Yoshizawa, 2019; Cao et al., 2024). Furthermore, we provide detailed morphological data via SEM imagery, including dorsal and ventral views (Fig. 2), magnified views of the pronotum and pteronotum (Fig. 4A and C), a specimen gripping feather fragments (Fig. 4B), and the detailed structure of the everted male genitalia (Fig. 4D). These SEM images provide foundational insights essential for understanding the detailed structures of *I*. *plataleae*.

The ML phylogenetic tree revealed that our two *I*. *plataleae* individuals clustered closely with another *I*. *plataleae* sequence with a high bootstrap value of 95.4%, providing robust molecular support for our morphological identification (Felsenstein, 1985) (Fig. 5). This phylogenetic analysis highlights two notable points. First, *Docophoroides brevis* (Dufour, 1835) nested within the genus *Ibidoecus* clade, appearing as a sister group to *I*. *plataleae* (Fig. 5). However, this placement may be an artifact of limited taxon sampling, rendering this specific relationship unreliable as the node received low support (<50% bootstrap). Secondly, despite *Anatoecus icterodes* (Nitzsch, 1818) being synonymized with *Anatoecus dentatus* (Scopoli, 1763) (Grossi et al., 2014), it is still presented as *A*. *icterodes* in our tree. This is because the *A*. *icterodes* sequence (ON643885) from Sweet et al. (2022) was deposited in GenBank without incorporating this synonymy. In conclusion, our phylogenetic analysis demonstrated that *A*. *icterodes* and *A*. *dentatus* formed a single clade with a high bootstrap value of 98.1%, strongly supporting their conspecificity (Fig. 5).

This study reports a new geographic distribution of *I*. *plataleae*, a chewing louse species parasitic on the internationally endangered Black-faced Spoonbill, which represents the first report of this species in the Republic of Korea. Although the current study identified only one species of chewing louse on the Black-faced Spoonbill, there is a high probability that other species exist in Korea. Given that *Eucolpocephalum femorale* and *Ardeicola plataleae* have been recorded on the Black-faced Spoonbill in Japan (Shimada and Yoshizawa, 2019) and considering the host's migratory habits (Kwon et al., 2015; Chen et al., 2021), discovering these species in Korea is highly likely. Consequently, further investigation of currently unrecorded chewing lice species on the Black-faced Spoonbill in Korea is warranted.

## Funding

This work was supported by grants from the 10.13039/501100005880National Institute of Biological Resources (NIBR202602201) and the 10.13039/100016214National Institute of Ecology (NIE-B-2025-46 for the capture and sample collection of Black-faced Spoonbill) of the Republic of Korea.

## CRediT authorship contribution statement

**Jeong Hun Shim:** Conceptualization, Data curation, Formal analysis, Methodology, Software, Visualization, Writing – original draft, Writing – review & editing. **Heon Woo Lee:** Methodology, Validation, Writing – review & editing. **Min-Seung Yang:** Investigation, Project administration, Writing – review & editing. **In-Ki Kwon:** Investigation, Project administration, Writing – review & editing. **Yeong-Deok Han:** Investigation, Methodology, Project administration, Supervision, Validation, Visualization, Writing – review & editing. **Seongjun Choe:** Conceptualization, Funding acquisition, Methodology, Project administration, Software, Supervision, Validation, Writing – review & editing.

## Conflict of interest

The authors declare no conflict.
